# Self-decisive algorithm for unconstrained optimization problems as in biomedical image analysis

**DOI:** 10.3389/fncom.2022.994161

**Published:** 2022-10-03

**Authors:** Farah Jaffar, Wali Khan Mashwani, Sanaa Mohammed Al-marzouki, Nudrat Aamir, Mohammad Abiad

**Affiliations:** ^1^Department of Mathematics, Shaheed Benazir Bhutto Women University, Peshawar, Pakistan; ^2^Institute of Numerical Sciences, Kohat University of Science and Technology, Kohat, Pakistan; ^3^Department of Statistics, Faculty of Sciences, King Abdulaziz University, Jeddah, Saudi Arabia; ^4^Department of Basic Sciences and Humanities, CECOS University of IT and Emerging Sciences, Peshawar, Pakistan; ^5^Department of Mathematics and Statistics, American University of the Middle East, Egaila, Kuwait

**Keywords:** quasi-Newton method, multi-step quasi-Newton method, medical informatics, image processing, covariance matrix, eigenvalues

## Abstract

This study describes the construction of a new algorithm where image processing along with the two-step quasi-Newton methods is used in biomedical image analysis. It is a well-known fact that medical informatics is an essential component in the perspective of health care. Image processing and imaging technology are the recent advances in medical informatics, which include image content representation, image interpretation, and image acquisition, and focus on image information in the medical field. For this purpose, an algorithm was developed based on the image processing method that uses principle component analysis to find the image value of a particular test function and then direct the function toward its best method for evaluation. To validate the proposed algorithm, two functions, namely, the modified trigonometric and rosenbrock functions, are tested on variable space.

## 1. Introduction

Imaging informatics plays a significant role in medical and engineering fields. In the diagnostic application software, during the segmentation procedure, different tools are used to interact with a visualized image and a graphical user interface (GUI) is used to parameterize the algorithms and for the visualization of multi-modal images and segmentation results in 2D and 3D. Hence, different toolkits, such as Medical Interaction Toolkit (Wolf et al., 2005) or the MevisLab (Ritter et al., 2011), are used to build appropriate GUIs, yielding an interface to integrate new algorithms from science to application. To produce better results, we used different sensors to generate a perfect image for denoising the tasks, thus focusing on massive denoising, as sometimes it is difficult for humans and computers to recognize the image. Furthermore, different filters (Wang, 2018) are used to get better denoising. The commonly used mathematical method for this purpose is the quasi-Newton method, which is preferred due to its better performance than other classical methods. Schröter and Sauer (2010) investigated quasi-Newton algorithms for medical image registration. Mannel and Rund (2021) implemented a hybrid semi-smooth quasi-Newton method for the non-smooth optimal control problems and proved its efficiency. Recently, Moghrabi et al. (2022) derived self-scaled quasi-Newton methods and proved their efficiency over the non-scaled version. Among the quasi-Newton methods, Broyden-Fletcher-Goldfarb-Shanno (BFGS) is a widely used method due to its better performance. Hence, this motivated the researchers to further develop these methods, one such method is the two-step quasi-Newton method.

The two-step quasi-Newton methods are considered to minimize unconstrained optimization problems.


(1) $$\begin{array}{l} \text{minimize}f\left(x\right),\text{ where }f:R^{n}\to R. \end{array}$$

The multi-step quasi-Newton methods, which were introduced by Ford and Moghrabi (1994, 1993), obtained the best results over the single-step quasi-Newton method. In the single-step quasi-Newton method, updation of Hessian approximation (*B*_*i*+1_) is required to satisfy the secant equation


(2) $$\begin{array}{l} B_{i+1}s_{i}=y_{i}, \end{array}$$

where ***s***_***i***_ is the step size in the variable space ***x***_***i***_


(3) $$\begin{array}{l} s_{i}=x_{i+1}-x_{i}, \end{array}$$

and ***y***_***i***_ is the step size in gradient space ***g*****(*****x***_***i***_**)**, such as


(4) $$\begin{array}{l} y_{i}=g\left(x_{i+1}\right)-g\left(x_{i}\right). \end{array}$$

The quasi-Newton equation must satisfy the true Hessian ***M***_***i*****+1**_ of Newton equation which is defined as follows:


(5) $$\begin{array}{l} M\left(x_{i+1}\right)\frac{dx\left(\tau *\right)}{d\tau }=\frac{dg\left(x\left(\tau *\right)\right)}{d\tau } \end{array}$$

In the case of the two-step quasi-Newton methods, the secant equation (2) is replaced by


(6) $$\begin{array}{l} B_{i+1}\left(s_{i}-\alpha_{i}s_{i-1}\right)=y_{i}-\alpha_{i}y_{i-1}, \end{array}$$

or


(7) $$\begin{array}{l} B_{i+1}r_{i}=w_{i}, \end{array}$$

which is derived by interpolating the quadratic curve *x*(τ) in variable space and *g*(τ) in gradient space


(8) $$\begin{array}{l} x\left(\tau_{k}\right)=x_{i-m+k+1},\;for\;k=0,1,2. \end{array}$$

(9) $$\begin{array}{l} g\left(x\left(\tau_{k}\right)\right)=g\left(x_{i-m+k+1}\right),\;for\;k=0,1,2. \end{array}$$

for the purpose of interpolating the Lagrange polynomial that is found suitable in Jaffar and Aamir (2020). Therefore,


(10) $$\begin{array}{l} x\left(\tau \right)=\overset{2}{\underset{k=0}{\sum }}L_{k}\left(\tau \right)x_{i+k-1}, \end{array}$$

(11) $$\begin{array}{l} g\left(x\left(\tau \right)\right)\approx \overset{2}{\underset{k=0}{\sum }}L_{k}\left(\tau \right)g\left(x_{i+k-1}\right), \end{array}$$

the derivatives of Equations (10) and (11) are defined as


(12) $$\begin{array}{l} \frac{dx\left(\tau_{2}\right)}{d\tau }\equiv r_{i} \end{array}$$

(13) $$\begin{array}{l} \frac{dgx\left(\tau_{2}\right)}{d\tau }\equiv w_{i},\text{and} \end{array}$$

the above relations obtained are substituted in Equation (14), which is a two-step form of Equation (5)


(14) $$\begin{array}{l} M\left(x_{i+1}\right)\frac{dx\left(\tau_{2}\right)}{d\tau }=\frac{dg\left(x\left(\tau_{2}\right)\right)}{d\tau } \end{array}$$

The new secant condition for the two-step quasi-Newton method is obtained in the form of Equation (6/7), which should be satisfied by the updated Hessian approximation ***B***_***i*****+1**_. The value of α_*i*_ in Equation (6) is given by


(15) $$\begin{array}{l} \alpha_{i}=\frac{\delta^{2}}{\left(2\delta +1\right)}, \end{array}$$

where


(16) $$\begin{array}{l} \delta =\frac{\left(\tau_{2}-\tau_{1}\right)}{\left(\tau_{1}-\tau_{0}\right)}. \end{array}$$

Hence, the Broyden-Fletcher-Goldfarb-Shanno (BFGS) formula for the two-step method is defined as


(17) $$\begin{array}{l} B_{i+1}=B_{i}+\frac{B_{i}r_{i}r_{i}^{T}B_{i}}{r_{i}^{T}B_{i}r_{i}}+\frac{w_{i}w_{i}^{T}}{r_{i}^{T}w_{i}} \end{array}$$

The standard Lagrange polynomial $L_{k}$ depends on the values of ${\left\{\tau_{k}\right\}}_{k=0}^{m}$ and is defined as


(18) $$\begin{array}{l} L_{k}\left(\tau \right)\equiv \overset{m}{\underset{j=0,j\neq k}{\prod }}\frac{\tau -\tau_{j}}{\tau_{k}-\tau_{j}}. \end{array}$$

The parametric values τ_*k*_, for k= 0, 1, 2....,m, used in the computation of vectors, ***r***_***i***_ and ***w***_***i***_, are found by the metric of the form


(19) $$\begin{array}{l} \phi_{N}\left(z_{1},z_{2}\right)={\left({\left(z_{1}-z_{2}\right)}^{T}N\left(z_{1}-z_{2}\right)\right)}^{\frac{1}{2}}. \end{array}$$

The matrix ***N*** is a positive definite matrix, and three choices are given for ***N*** as ***I***, ***B***_***i***_, and ***B***_***i*****+1**_ on variable space and *z*_1_, *z*_2_ ϵ *R*^*n*^.

This metric between different iterates in the current interpolation is measured by fixed-point and accumulative approaches (Ford and Moghrabi, 1994). In this study, an accumulative approach is used for finding the parametric values.

• **Accumulative Methods**

These methods accumulate the distance between the consecutive iterates in their natural sequence. The latest iterate ***x***_*i*+1_, corresponding to the value τ_*m*_ of τ, is considered as the origin or base point, and the other values of τ are calculated by accumulating the distance between the consecutive pairs. Therefore, we have


(20) $$\begin{array}{l} \tau_{m}=0, \end{array}$$

(21) $$\begin{array}{l} \tau_{k}=\tau_{k+1}-\phi_{N}\left(x_{i-m+k+2},x_{i-m+k+1}\right),\text{for }k=0,1,....m-1. \end{array}$$

In the two-step method, the accumulative type is determined as A1, A2, and A3, and the parametric values are found with the help of Equation (21) for k=0,1, where the base point will be τ_2_ = 0 for m=2 from Equation (20).

• **Algorithm A1**

The identity matrix ***I*** is taken as matrix ***N*** in this algorithm.


$$\begin{array}{l} \tau_{1}=\tau_{2}-\phi_{I}\left(x_{i-2+1+2},x_{i-2+1+1}\right), \end{array}$$

(22) $$\begin{array}{l} =-\phi_{I}\left(x_{i+1},x_{i}\right)=-||s_{i}{||}_{2}, \end{array}$$

$$\begin{array}{l} \tau_{0}=\tau_{1}-\phi_{I}\left(x_{i},x_{i-1}\right), \end{array}$$

(23) $$\begin{array}{l} =-||s_{i}{||}_{2}-||s_{i-1}{||}_{2}. \end{array}$$

• **Algorithm A2**

In this algorithm, matrix ***N*** is taken as the current Hessian approximation ***B***_*i*_.


$$\begin{array}{l} \tau_{1}=\tau_{2}-\phi_{B_{i}}\left(x_{i+1},x_{i}\right), \end{array}$$

(24) $$\begin{array}{l} \Rightarrow \tau_{1}=\sqrt{s_{i}^{T}B_{i}s_{i}}, \end{array}$$

The above equation involves a matrix vector product which is computationally expensive. For instance, with the help of search direction, we can easily compute the parameters of the same situation as


(25) $$\begin{array}{l} p_{i}=-B_{i}^{-1}g\left(x_{i}\right), \end{array}$$

since


$$\begin{array}{l} x_{i+1}=x_{i}+t_{i}p_{i} \end{array}$$

$$\begin{array}{l} =x_{i}-t_{i}B_{i}^{-1}g\left(x_{i}\right)\text{ for }t_{i}>0, \end{array}$$

$$\begin{array}{l} x_{i+1}-x_{i}=-t_{i}B_{i}^{-1}g\left(x_{i}\right), \end{array}$$

$$\begin{array}{l} s_{i}=-t_{i}B_{i}^{-1}g\left(x_{i}\right), \end{array}$$

(26) $$\begin{array}{l} \Rightarrow B_{i}s_{i}=-t_{i}g\left(x_{i}\right), \end{array}$$

By substituting Equation (26) in Equation (24), we get


(27) $$\begin{array}{l} \tau_{1}=-\sqrt{t_{i}s_{i}^{T}g\left(x_{i}\right)}, \end{array}$$

The above expression is easy to calculate but the expression $s_{i-1}^{T}B_{i}s_{i-1}$ in τ_0_ is very difficult to compute in every iteration. Therefore, to lessen the computational cost, Ford and Moghrabi (1994) claimed that, in multi-step methods, when the quasi-Newton equation in Equation (2) is not satisfied, then we can consider that it satisfies approximately by replacing i+1 with i in Equation (2), where ***B***_*i*_ is an approximation of matrix N. Therefore, we obtain


(28) $$\begin{array}{l} B_{i}s_{i-1}\approx y_{i-1} \end{array}$$

$$\begin{array}{l} \tau_{0}=\tau_{1}-\phi_{B_{i}}\left(x_{i},x_{i-1}\right),\text{and} \end{array}$$

using Equation (28), we have


$$\begin{array}{l} \tau_{0}=\sqrt{-t_{i}s_{i}^{T}g\left(x_{i}\right)}-\sqrt{s_{i-1}^{T}y_{i-1}}. \end{array}$$

• **Algorithm A3**

In this algorithm, the choice of matrix ***N*** is ***B***_***i*****+1**_, which is the Hessian approximation at ***x***_***i*****+1**_.


$$\begin{array}{l} \tau_{1}=\tau_{2}-\phi_{B_{i+1}}\left(x_{i+1},x_{i}\right), \end{array}$$

(29) $$\begin{array}{l} \tau_{1}=-\sqrt{s_{i}^{T}y_{i}}, \end{array}$$

$$\begin{array}{l} \tau_{0}=\tau_{1}-\phi_{B_{i+1}}\left(x_{i},x_{i-1}\right), \end{array}$$

(30) $$\begin{array}{l} \tau_{0}\approx -\sqrt{s_{i}^{T}y_{i}}-\sqrt{s_{i}^{T}y_{i-1}}, \end{array}$$

Since τ_1_ and τ_0_ are expensive to compute, Equation (2) is used.

Aamir and Ford (2021) investigated the multi-step skipping technique in which one-step and two-step skipping strategies were experimented and produced better results than those without skipping strategy. The authors also modified the search direction, which was implemented with/without the skipping technique to achieve good performance in minimum time duration. We experimented two test functions of different dimensions by the two-step quasi-Newton methods with different techniques, i.e., one-step skipping with no modified search direction and one-step skipping with modified search direction on variable space with a high rate of computational effort. Therefore, to lessen the computational burden and increase efficiency, an algorithm was developed to execute a particular function by using the best method only. Section 2 discusses the two-step quasi-Newton method with different techniques in detail. Section 3 proposes a self-decisive algorithm which is developed based on image processing method to find the image values of different test functions. Section 4 discusses the experimental setup of the proposed strategy. Section 5 analyzes the numerical results of one function, which can help the algorithm to execute a particular function by the best method only. In the last section, the conclusion is drawn based on different numerical simulations.

## 2. Two-step quasi-Newton methods with different techniques

Different techniques in two-step methods, such as the one-step skipping technique with no modified search direction and the one-step skipping technique with modified search direction, are implemented on the selected test functions for the purpose of minimization. These functions are examined by function evaluation, the number of iterations, and time in seconds. The notation of different methods on different techniques is given in Table 1.

**Table 1:** Notation of methods with different techniques.

**Notation**	**Discription**
${\;}_{\left[0\right]}^{\left[1\right]}{\text{A}}_{n}^{\left(2\right)}$	Accumulative two-step method with one update skipped
	and no modified search direction
${\;}_{\left[1\right]}^{\left[1\right]}{\text{A}}_{n}^{\left(2\right)}$	Accumulative two-step method with one update skipped
	and modified search direction
	**where n=1,2,3 corresponds to matrices *I, B*_*i*_, *B*_*i*+1_ respectively on variable space**

### 2.1. Skipping technique

In quasi-Newton methods, updation of Inverse Hessian approximation ***H***_***i***_ to ***H***_***i*****+1**_ is a very expensive procedure under certain circumstances. Tamara et al. (1998) introduced the idea of skipping updates for certain steps to lessen the burden of computational cost. They investigated the question of “how much and which information can be dropped in BFGS and other quasi-Newton methods without destroying the property of quadratic termination” and called this procedure “backing up.” They used this idea in the algorithm if the step length is 1.0 or the current iteration is odd.

Aamir and Ford (2021) investigated the skipping technique in single step and multi-step methods. The experimental results of comparison between the skipping and non-skipping methods revealed that skipping algorithms outperformed non-skipping algorithms.

• **Algorithm of the Multi-step Skipping Method**

The general algorithm of the skipping technique is as follows:

1. Select an initial approximation ***x***_**0**_ and ***H***_**0**_ and set i=1
2. For j=1: m (where m is the number of steps to be skipped)
  - Calculate a search direction ***p***_***i*****+*j*−2**_ **= −*****H***_***i*****−1**_***g***_***i*****+*****j*****−2**_.
  - Find t by giving *t*_*i*+*j*−2_ for executing line search ***x***_*i*+*j*−2_+*t**p***_*i*+*j*−2_.
  - Calculate new approximation ***x***_***j***_ **=** ***x***_***j***_+*t*_*j*_***p***_*j*_.

End for

3. By the use of different methods, update ***H***_***i*****+*****j*****−2**_ to give ***H***_***i*****+*****m*****−1**_.
4. If ||***g***_*i*_|| ≤ ϵ, then stop, else i:=i+m and go to step 2. End if.

• **Application of the skipping technique on the two-step method**

Now that we are at ***x***_***i*****+1**_, the matrix is updated by ***B***_***i*****−1**_**,** ***s***_***i***_**,** ***s***_***i*****−1**_**,** ***y***_***i***_, and ***y***_***i*****−1**_, using the following steps:

1. Using the above terms, compute τ_*k*_ and then find δ.

2. By the use of all the above values, through which we find ***r***_***i***_ and ***w***_***i***_, we have


(31) $$\begin{array}{l} r_{i}=s_{i}-\frac{\delta^{2}}{\left(2\delta +1\right)}s_{i-1}, \end{array}$$

(32) $$\begin{array}{l} w_{i}=y_{i}-\frac{\delta^{2}}{\left(2\delta +1\right)}y_{i-1}, \end{array}$$

3. The Hessian approximation is updated by using all the above values.


(33) $$\begin{array}{l} B_{i+1}=BFGS\left(B_{i-1},r_{i},w_{i}\right). \end{array}$$

Now we compute τ_*k*_ and/or δ under different methods.

### 2.2. The two-step method with skipping and modified search directions

Here, we explained the derivation of the modified search direction. The following notations are used during the derivation of modified search direction in the skipping technique.

***Ĥ***_***i***_ represents that the matrix is never computed.

${\tilde{p}}_{i}$ represents modified search direction.

Now, let us consider that the single-step BFGS updated the matrix ***H***_***i*****−1**_. The search direction ***p***_***i*****−1**_ is defined as


(34) $$\begin{array}{l} p_{i-1}=-H_{i-1}g_{i-1}. \end{array}$$

By using the skipping technique, the next search direction is


(35) $$\begin{array}{l} p_{i}=-H_{i-1}g_{i}. \end{array}$$

With the help of ***Ĥ***_***i***_, we can find the modified search direction ${\tilde{p}}_{i}$. We define


$$\begin{array}{l} {\hat{H}}_{i}=BFGS\left(H_{i-1},s_{i-1},y_{i-1}\right) \end{array}$$

(36) $$\begin{array}{l} =H_{i-1}+\lambda_{i-1}s_{i-1}s_{i-1}^{T}-\alpha_{i-1}\left(H_{i-1}y_{i-1}s_{i-1}^{T}+s_{i-1}y_{i-1}^{T}H_{i-1}\right), \end{array}$$

where


$$\begin{array}{l} \lambda_{i-1}=\left(1+\frac{y_{i-1}^{T}H_{i-1}y_{i-1}}{s_{i-1}^{T}y_{i-1}}\right)\frac{1}{s_{i-1}^{T}y_{i-1}} \end{array}$$

$$\begin{array}{l} \alpha_{i-1}=\frac{1}{s_{i-1}^{T}y_{i-1}} \end{array}$$

Now, the modified search direction is


$$\begin{array}{l} {\tilde{p}}_{i}=-{\hat{H}}_{i}g_{i} \end{array}$$

$$\begin{array}{l} =H_{i-1}g_{i}+\lambda_{i-1}s_{i-1}\left[s_{i-1}^{T}g_{i}\right]\\ \;+\alpha_{i-1}\left(H_{i-1}y_{i-1}\left[s_{i-1}^{T}g_{i}\right]+s_{i-1}\left[y_{i-1}^{T}H_{i-1}g_{i}\right]\right), \end{array}$$

and by Equation (35), we get


(37) $$\begin{array}{l} =p_{i}+\lambda_{i-1}\left[s_{i-1}^{T}g_{i}\right]s_{i-1}+\alpha_{i-1}\left([s_{i-1}^{T}g_{i}\right]H_{i-1}y_{i-1}\\ \;+\left[y_{i-1}^{T}p_{i}\right]s_{i-1}), \end{array}$$

From the above equation, it can be observed that ***H***_***i*****−1**_***y***_***i*****−1**_ and λ_*i*−1_ cannot be easily computable due to the matrix vector product than other terms. However, with the help of Equation (34) and Equation (35), the expression ***H***_***i*****−1**_***y***_***i*****−1**_ can be defined as


$$\begin{array}{l} H_{i-1}y_{i-1}=H_{i-1}\left(g_{i}-g_{i-1}\right) \end{array}$$

(38) $$\begin{array}{l} =-p_{i}+p_{i-1} \end{array}$$

Therefore, using the above equation in Equation (37), modified search direction can be calculated efficiently without explicitly computing ${\hat{H}}_{i}$.

• **Algorithm of the Multi-step Skipping Quasi-Newton method with Modified Search Direction**

The general algorithm is given as follows:

1. Select ***x***_**0**_ and ***H***_**0**_ as an initial approximation; set i=1
2. For j=1:m, where m is the number of steps,
3. Calculate ***p***_***i*****+*****k*****−2**_ **= −*****H***_***i*****−1**_***g***_***i*****+***k***−2**_.
4. Calculate modified search direction ${\tilde{p}}_{i+k-2}=-{Ĥ}_{i+j-2}g_{i+k-2}$.
5. Do the line search along ***x***_***i*****+*****j*****−2**_+*t**p***_***i*****+*****j*****−2**_ and also providing a value of *t*_*i*+*j*−2_ for t.
6. Calculate new approximation $x_{j}=x_{j}+t_{j}\tilde{p_{i}}$.End
7. Update ***H***_***i*****−1**_ to produce ***H***_***i*****+*****m*****−1**_ by using different methods discussed in previous sections.
8. Check for convergence, if it is not converged, then i=i+1 and go to step no: 2.

## 3. Image processing

In the viewpoint of image processing “an image is an array or matrix of numeric values called pixels (Picture Element) arranged in columns and rows”. In mathematics an image is defined as “a graph of a spatial function” or “it is a two-dimensional function f(x,y), where x and y are the spatial (plane) coordinates, and the amplitude at any pair of coordinates (x,y) is called the intensity of the image at that level.” If x,y and the amplitude values of f are finite and discrete quantities, we call the image a digital image. A digital image is composed of a finite number of pixels, each of which has a particular location and value. Image processing is a process in which different mathematical operations are performed subject to application on the image to get improved or to extract significant information from the image for subsequent processing. When this process is applied to digital images is called digital image processing.

Digital image processing has a wide scope for researchers to work on various areas of science (such as, a agriculture, biomedical, and engineering). Previous studies showed that researchers applied and investigated different techniques of image processing for analysis and problem solving, such as detection and measurement of paddy leaf disease symptoms using image processing (Narmadha and Arulvadivu, 2017), breast cancer detection using image processing techniques (Christian et al., 2000), a novel outlier detection method for monitoring data in dam engineering (Shao et al., 2022), and counterfeit electronics detection using image processing and machine learning (Navid et al., 2017).

### 3.1. Proposed strategy

**Figure 1:**
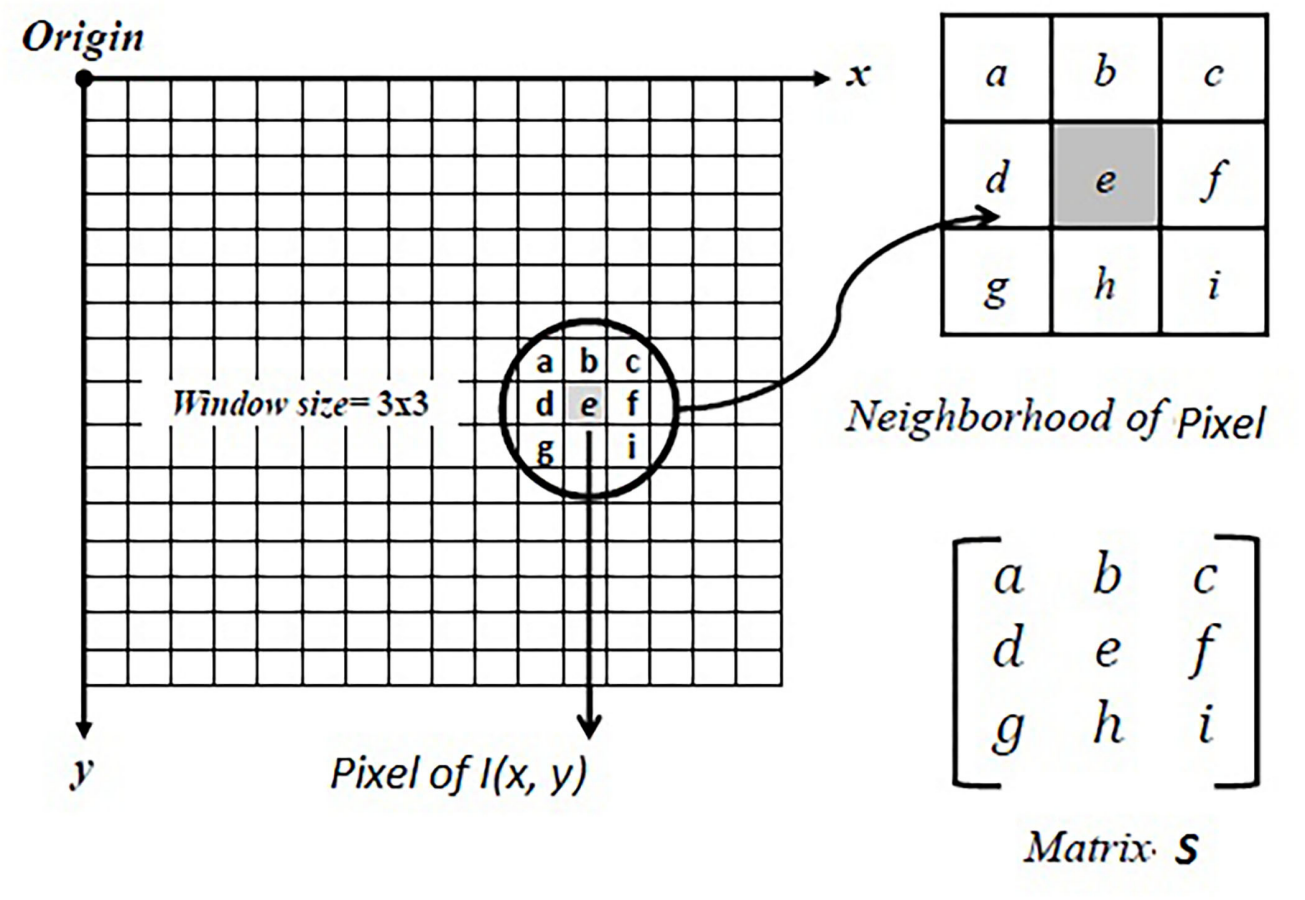
Generated window denoted as matrix S.

In the proposed strategy, the algorithm is developed by which the image values of different images I(x, y) of test functions are obtained by statistical technique, and the desired objective is achieved. In the first step, the images of different test functions are obtained with a resolution of 600 × 600 pixels. In the second step, the window of a size *W*×*W* is generated around each pixel of the image I(x, y), where the suggested size of the generated window is 3 × 3 and this window is treated as matrix S. The rows of the matrix S are considered as observations, and columns are considered as variables. Figure 1 shows the schematic diagram of the matrix S generation.

In the third step, covariance matrix *C*^(*x, y*)^ of matrix S is computed with the help of the following equation:


(39) $$\begin{array}{l} C^{\left(x,y\right)}=\frac{1}{W-1}\left[S^{t}S\right] \end{array}$$

In the fourth step, eigenvalues of the covariance matrix are calculated. The sum of the eigenvalues is directly proportional to edge strength, which is calculated as follows:


(40) $$\begin{array}{l} EdgeStrength=\overset{W}{\underset{k=1}{\sum }}eigen_{k}ofC_{h}^{\left(x,y\right)} \end{array}$$

The third and fourth steps are done twice, the first time for the horizontal edge strength and the second time for the vertical edge strength calculation. Therefore, Equations (39) and (40) are used for calculating horizontal and vertical edge strength generation as follows:


(41) $$\begin{array}{l} C_{h}^{\left(x,y\right)}=\frac{1}{W-1}\left[S^{t}S\right] \end{array}$$

(42) $$\begin{array}{l} C_{v}^{\left(x,y\right)}=\frac{1}{W-1}\left[S^{t}S\right] \end{array}$$

(43) $$\begin{array}{l} EdgeStrength_{h}=\overset{W}{\underset{k=1}{\sum }}eigen_{k}ofC_{h}^{\left(x,y\right)} \end{array}$$

(44) $$\begin{array}{l} EdgeStrength_{v}=\overset{W}{\underset{k=1}{\sum }}eigen_{k}ofC_{v}^{\left(x,y\right)} \end{array}$$

The sum of horizontal and vertical edge strength gives the value of a pixel of I(x, y). Hence, the value of each pixel of an image is calculated as


(45) $$\begin{array}{l} V\left(x,y\right)=\left(EdgeStrength_{h}\right)+\left(EdgeStrength_{v}\right),\text{and} \end{array}$$

the sum of all pixel values gives the value of an image I(x, y) defined as


(46) $$\begin{array}{l} V\left(I\right)=\overset{n}{\underset{i=1}{\sum }}V\left(x,y\right) \end{array}$$

## 4. Experimental setup and results

Two test functions were selected from literature and were executed by using different techniques of the two-step quasi-Newton methods on variable space. The execution of test functions by every technique was computationally expensive. Therefore, an algorithm is required to enable the researchers to execute a particular function by the best method only to reduce computational cost.

Hence, our objective is to develop such an algorithm that can compute the image value of every input image of the test function and forward each function to the method by which it outperformed. The algorithm works in the following steps:

1. Obtain the images of each test function.
2. Compute the image value of each image.
3. Classify test functions by their image values.
4. Forward the particular function toward the best method.

### 4.1. Test functions

To check the performance of different techniques used in two-step methods, we considered two test functions of different dimensions with four different starting points and epsilon value from the literature (Hillstrom et al., 1981), which are reported in Tables 2, 3. These test functions are categorized into three classes, namely soft, medium, and hard.

1. Soft: (2 ≤ *n* ≤ 20);
2. Medium: (20 ≤ *n* ≤ 60);
3. Hard: (61 ≤ *n* ≤ 150);
4. Combined: (2 ≤ *n* ≤ 150).

**Table 2:** Test problem and dimensions in different test sets.

		**Starting points**			
**Function Name and dimension**		**[a]**	**[b]**	**[c]**	**[d]**
**Rosenbrock**			**Soft**		
(2)	e = 10^−7^	(-1.2, 1.0)	(-120, 100)	(20, -20)	(6.39, -0.221)
(20)	e = 10^−7^	([-1.2, 1.0])	(1, 2,....,20)	([6.39, -0.221])	(-1, -1, -1, -1, -1, 1,....1)
			**Medium**		
(26)	e = 10^−7^	([-1.2, 1.0])	([F])	([20])	([6.39, -0.221])
(40)	e = 10^−7^	([-1.2, 1])	([-120, 100])	([1, -2, 3, -4,...., -10])	([20])
(60)	e = 10^−7^	([-1.2, 1])	([F])	([F])	([6.39, -0.221])
			**Hard**		
(80)	e= 10^−7^	([-1.2, -1.0])	([F])	([F])	([F])
(100)	e=10^−7^	([-1.2, -1])	([F])	([F])*([F])	
(120)	e=10^−7^	([-1.2, -1])	([20])	[F]	([6.39, -0.221])

**Table 3:** Test problem and dimensions in different test sets.

		**Starting points**			
**Function name and dimension**		**[a]**	**[b]**	**[c]**	**[d]**
**Modified Trigonometric**			**Soft**		
(16)	e = 10^−7^	([–2, –1, 1, 2])	([–2, 1.5,.., –1.5, 2])	([0.1, 1, –0.1, –1])	([2.5, 2, 1.5, 1, 0.5], 2.5)
			**Medium**		
(32)	e = 10^−7^	([–2, –1, 1, 2]	([–2, 1.5,.., –1.5, 2 ]	([0.1, 1, –0.1, 1]	([2.5, 2,..,0.5], 2.5, 2)
(64)	e = 10^−6^	([–2, –1, 1, 2])	([–2, 1.5,..., –1.5, 2])	([0.1, 1, –0.1, 1])	([2.5, 2,.. 0.5], 2.5,.., 1)
			**Hard**		
(95)	e= 10^−5^	([–2, –1, 1, 2],–2, –1, 1)	([–2, 1.5,.., 2])	([0.1, 1.0, –0.1, 1.0])	([2.5, 2.0, 1.5, 1.0, 0.5])
(128)	e= 10^−6^	([–2, –1, 1, 2], –2, –1, 1)	([–2, 1.5,.., –1.5, 2])	([0.1, 1, –0.1, 1])	([2.5, 2, 1.5, 1, 0.5], 2.5, 2, 1.5)
(150)	e= 10^−5^	([–2, –1, 1, 2], –2, –1)	([–2, 1.5, ..., 2], –2,..., –0.5, 1)	([0.1, 1, –0.1, 1], 0.1, 1)	([2.5, 2.0, 1.5, 1.0, 0.5])

**Figure 2:**
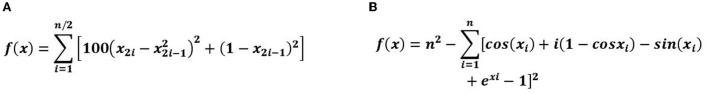
Images of test functions. **(A)** I1; **(B)** I2.

The equations of both test functions are mentioned below by which 600 × 600 resolution images (displayed in Figure 2) are generated to calculate the image value of each function (as reported in Table 4) and which are programmed successfully in the self-decisive algorithm.

1. Extended Rosenbrock function:
  $$\begin{array}{l} f\left(x\right)=\overset{n/2}{\underset{i=1}{\sum }}[100{\left(x_{2i}-x_{2i-1}^{2}\right)}^{2}+{\left(1-x_{2i-1}\right)}^{2}] \end{array}$$
2. Modified Trigonometric function:
  $$\begin{array}{l} f\left(x\right)=n^{2}-\overset{n}{\underset{i=1}{\sum }}[cos\left(x_{i}\right)+i\left(1-cosx_{i}\right)-sin\left(x_{i}\right)+e^{x_{i}}-1{]}^{2}] \end{array}$$

**Table 4:** Image values of test functions.

**Image**	**Test functions**	**Value**
I1	Extended rosenbrock	0.1009
I2	Modified trigonometric	0.1004

### 4.2. Self decisive algorithm

An outline of the self decisive algorithm can be defined as follows:

**Step 0:** Obtain image I(x, y) to generate window/matrix S.

**Step 1:** Compute covariance matrix $C^{\left(x,y\right)}=\frac{1}{W-1}\left[S^{t}S\right]$.

**Step 2:** Find eigenvalues of *C*^(*x, y*)^.

**Step 3:** Calculate the value of each pixel V(x, y)= (strength h)+ (strength v).

**Step 4:** Compute the value of each image $V\left(I\right)=\overset{n}{\underset{i=1}{\sum }}V\left(x,y\right)$.

**Step 5:** Set threshold on V(I).

**Step 6:** Function execution by indicated/best method type.

## 5. Numerical analysis of test functions

Two test functions, namely, Rosenbrock and modified trigonometric functions, are selected from the literature (Hillstrom et al., 1981) to evaluate their performance by using two different two-step techniques, i.e., one-step skipping with no modified search direction and one-step skipping with modified search direction. These functions are of different dimensions ranging from 2 to 150.

### 5.1. Discussion on rosenbrock function

- It is evident from Table 5 that the function outperformed by the method ${(}_{\text{[0]}}^{\text{[1]}}{\text{A}}_{\text{1}}^{\text{(2)}}$) during function evaluation and number of iterations, and time reduction is noted by ${(}_{\text{[0]}}^{\text{[1]}}{\text{A}}_{\text{1}}^{\text{(2)}}$) in soft dimension. In medium dimension, the ${(}_{\text{[0]}}^{\text{[1]}}{\text{A}}_{\text{2}}^{\text{(2)}}$) and ${(}_{\text{[0]}}^{\text{[1]}}{\text{A}}_{\text{2}}^{\text{(3)}}$) methods outperformed in function evaluation, the number of iterations, respectively, while the ${(}_{\text{[0]}}^{\text{[1]}}{\text{A}}_{\text{1}}^{\text{(2)}}$) method exhibited reduction in time. In hard dimension, the ${(}_{\text{[0]}}^{\text{[1]}}{\text{A}}_{\text{2}}^{\text{(2)}}$) method showed better results to minimize the function evaluation and the number of iterations and the ${(}_{\text{[0]}}^{\text{[1]}}{\text{A}}_{\text{1}}^{\text{(2)}}$) method reduced the computational effort.
- The results of Table 6 show that the ${(}_{\text{[1]}}^{\text{[1]}}{\text{A}}_{\text{1}}^{\text{(2)}}$) method outperformed in terms of function evaluation, the number of iterations, and computational time in soft dimension problems. In medium dimension, the ${(}_{\text{[1]}}^{\text{[1]}}{\text{A}}_{\text{2}}^{\text{(2)}}$) method exhibited best results in function evaluation, the number of iterations, and computational time. The ${(}_{\text{[1]}}^{\text{[1]}}{\text{A}}_{\text{2}}^{\text{(2)}}$) method showed a reduction in function evaluation and the number of iterations, and the ${(}_{\text{[1]}}^{\text{[1]}}{\text{A}}_{\text{1}}^{\text{(2)}}$) method is executed in less time.

**Table 5:** Results of rosenbrock function of all dimension problems in a two-step method of the first technique.

**Method**	**Function** **Eval**	**Iteration**	**Time in sec**	**Failure**	**Dimension**
${\;}_{\left[0\right]}^{\left[1\right]}A_{1}^{\left(2\right)}$	**60**	**43**	0.0243	0	
${\;}_{\left[0\right]}^{\left[1\right]}A_{2}^{\left(2\right)}$	62	45	0.0309	0	Soft
${\;}_{\left[0\right]}^{\left[1\right]}A_{3}^{\left(2\right)}$	62	45	**0.0205**	0	
${\;}_{\left[0\right]}^{\left[1\right]}A_{1}^{\left(2\right)}$	65	46	**0.0180**	0	
${\;}_{\left[0\right]}^{\left[1\right]}A_{2}^{\left(2\right)}$	**44**	46	0.0256	0	Medium
${\;}_{\left[0\right]}^{\left[1\right]}A_{3}^{\left(2\right)}$	65	**44**	0.0453	0	
${\;}_{\left[0\right]}^{\left[1\right]}A_{1}^{\left(2\right)}$	65	46	**0.0307**	0	
${\;}_{\left[0\right]}^{\left[1\right]}A_{2}^{\left(2\right)}$	**65**	**44**	0.0449	0	Hard
${\;}_{\left[0\right]}^{\left[1\right]}A_{3}^{\left(2\right)}$	65	44	0.0629	0	

The bold values indicate the good experimental results provided by the proposed methods in terms of function evaluation, number of iterations and time elapsed in seconds, which is one of our objectives in the study of this paper.

**Table 6:** Results of rosenbrock function of all dimension problems in the two-step method of the second technique.

**Method**	**Function** **Eval**	**Iteration**	**Time in sec**	**Failure**	**Dimension**
${\;}_{\left[1\right]}^{\left[1\right]}A_{1}^{\left(2\right)}$	**62**	**30**	**0.0283**	0	
${\;}_{\left[1\right]}^{\left[1\right]}A_{2}^{\left(2\right)}$	62	34	0.0330	0	Soft
${\;}_{\left[1\right]}^{\left[1\right]}A_{3}^{\left(2\right)}$	62	31	0.0423	0	
${\;}_{\left[1\right]}^{\left[1\right]}A_{1}^{\left(2\right)}$	51	44	0.0230	0	
${\;}_{\left[1\right]}^{\left[1\right]}A_{2}^{\left(2\right)}$	**49**	**39**	**0.0158**	0	Medium
${\;}_{\left[1\right]}^{\left[1\right]}A_{3}^{\left(2\right)}$	49	39	0.0193	0	
${\;}_{\left[1\right]}^{\left[1\right]}A_{1}^{\left(2\right)}$	51	44	**0.0298**	0	
${\;}_{\left[1\right]}^{\left[1\right]}A_{2}^{\left(2\right)}$	**49**	**39**	0.0309	0	Hard
${\;}_{\left[1\right]}^{\left[1\right]}A_{3}^{\left(2\right)}$	49	39	0.0405	0	

The bold values indicate the good experimental results provided by the proposed methods in terms of function evaluation, number of iterations and time elapsed in seconds, which is one of our objectives in the study of this paper.

• **Comparative analysis of both techniques**

- The behavior of both techniques was compared, and our analysis concluded that one-step skipping with no modified search direction outperformed in function evaluation and computational time, while the second technique, i.e., one-step skipping with modified search direction, showed a reduction in the number of iterations in all dimension problems.

### 5.2. Discussion on modified trigonometric function

- Table 7 shows the function outperformed by the ${(}_{\left[\text{0}\right]}^{\left[\text{1}\right]}{\text{A}}_{\text{1}}^{\left(\text{2}\right)})$ method in function evaluation, the number of iterations, and in computational time in soft dimension. In medium dimension, the ${(}_{\left[\text{0}\right]}^{\left[\text{1}\right]}{\text{A}}_{\text{2}}^{\left(\text{2}\right)})$ method outperformed in function evaluation and the number of iteration while less computational time is noted by the ${(}_{\left[\text{0}\right]}^{\left[\text{1}\right]}{\text{A}}_{\text{1}}^{\left(\text{2}\right)})$ method. The ${(}_{\left[\text{0}\right]}^{\left[\text{1}\right]}{\text{A}}_{\text{3}}^{\left(\text{2}\right)})$ method showed better results in function evaluation, while the ${(}_{\left[\text{0}\right]}^{\left[\text{1}\right]}A_{2}^{\left(\text{2}\right)})$ and ${(}_{\left[\text{0}\right]}^{\left[\text{1}\right]}A_{\text{1}}^{\left(\text{2}\right)})$ methods outperformed in the number of iterations and time, respectively, in hard dimension.
- In Table 8, the ${(}_{\left[\text{1}\right]}^{\left[\text{1}\right]}{\text{A}}_{\text{2}}^{\left(\text{2}\right)})$ method showed the best results in function evaluation and the number of iterations in soft and medium dimensions, and the time reduction is observed by the ${(}_{\left[\text{1}\right]}^{\left[\text{1}\right]}{\text{A}}_{\text{1}}^{\left(\text{2}\right)})$ method in soft dimension and by the ${(}_{\left[\text{1}\right]}^{\left[\text{1}\right]}{\text{A}}_{\text{2}}^{\left(\text{2}\right)})$ method in medium dimension. In hard dimension, the ${(}_{\left[\text{1}\right]}^{\left[\text{1}\right]}{\text{A}}_{\text{1}}^{\left(\text{2}\right)})$ method exhibited minimum results in function evaluation and computational time, and the ${(}_{\left[\text{1}\right]}^{\left[\text{1}\right]}{\text{A}}_{\text{3}}^{\left(\text{2}\right)})$ method showed a reduction in the number of iterations.

**Table 7:** Results of modified trigonometric function of all dimension problems in the two-step method of the first technique.

**Method**	**Function** **Eval**	**Iteration**	**Time in sec**	**Failure**	**Dimension**
${\;}_{\left[0\right]}^{\left[1\right]}A_{1}^{\left(2\right)}$	**104**	**75**	**0.0312**	0	
${\;}_{\left[0\right]}^{\left[1\right]}A_{2}^{\left(2\right)}$	144	95	0.0415	0	Soft
${\;}_{\left[0\right]}^{\left[1\right]}A_{3}^{\left(2\right)}$	144	95	0.0590	0	
${\;}_{\left[0\right]}^{\left[1\right]}A_{1}^{\left(2\right)}$	170	111	**0.0500**	0	
${\;}_{\left[0\right]}^{\left[1\right]}A_{2}^{\left(2\right)}$	**168**	**111**	0.0521	0	Medium
${\;}_{\left[0\right]}^{\left[1\right]}A_{3}^{\left(2\right)}$	168	112	0.0705	0	
${\;}_{\left[0\right]}^{\left[1\right]}A_{1}^{\left(2\right)}$	199	127	**0.1747**	0	
${\;}_{\left[0\right]}^{\left[1\right]}A_{2}^{\left(2\right)}$	193	**125**	0.2117	0	Hard
${\;}_{\left[0\right]}^{\left[1\right]}A_{3}^{\left(2\right)}$	**175**	128	0.2160	0	

The bold values indicate the good experimental results provided by the proposed methods in terms of function evaluation, number of iterations and time elapsed in seconds, which is one of our objectives in the study of this paper.

**Table 8:** Results of modified trigonometric function of all dimension problems in the two-step method of the second technique.

**Method**	**Function** **Eval**	**Iteration**	**Time in sec**	**Failure**	**Dimension**
${\;}_{\left[1\right]}^{\left[1\right]}A_{1}^{\left(2\right)}$	110	109	**0.0400**	0	
${\;}_{\left[1\right]}^{\left[1\right]}A_{2}^{\left(2\right)}$	**108**	**103**	0.0544	0	Soft
${\;}_{\left[1\right]}^{\left[1\right]}A_{3}^{\left(2\right)}$	109	104	0.0408	0	
${\;}_{\left[1\right]}^{\left[1\right]}A_{1}^{\left(2\right)}$	137	132	0.0544	0	
${\;}_{\left[1\right]}^{\left[1\right]}A_{2}^{\left(2\right)}$	**136**	**132**	**0.0516**	0	Medium
${\;}_{\left[1\right]}^{\left[1\right]}A_{3}^{\left(2\right)}$	136	132	0.0568	0	
${\;}_{\left[1\right]}^{\left[1\right]}A_{1}^{\left(2\right)}$	**147**	146	**0.1808**	0	
${\;}_{\left[1\right]}^{\left[1\right]}A_{2}^{\left(2\right)}$	147	145	0.1895	0	Hard
${\;}_{\left[1\right]}^{\left[1\right]}A_{3}^{\left(2\right)}$	147	**145**	0.1869	0	

The bold values indicate the good experimental results provided by the proposed methods in terms of function evaluation, number of iterations and time elapsed in seconds, which is one of our objectives in the study of this paper.

• **Comparative analysis of both techniques**

- Both techniques were compared and analyzed based on experimental results. From the analysis, it can be concluded that one-step skipping with no modified search direction outperformed in function evaluation, the number of iterations, and computational time, except the one case of medium dimension, in which the second technique, i.e., one-step skipping with modified search direction, showed a reduction in function evaluation.

## 6. Conclusion

An algorithm was developed to compute the image value of a particular test function and direct it to its best method for execution. The two-step quasi-Newton methods with two techniques (one-step skipping with no modified search direction and one-step skipping with modified search direction) were chosen and experimented on two test functions, namely, Rosenbrock and modified trigonometric function. The best method was determined using the experimental results obtained in terms of function evaluation, the number of iterations, and computational time. This study concluded that the one-step skipping without modification in search direction technique showed superiority over the one-step skipping with modified search direction technique under both test functions. Hence, this algorithm directed all the functions having the same image value as Rosenbrock and modified trigonometric functions to the one-step skipping technique with no modified search direction.

## 7. Future work

To further strengthen the algorithm reported in this study, we propose to investigate the image recognition in terms of picture or graph instead of image value and then direct the reported function (or medical image) to the best method available for the obtaining solution. Based on the literary research, in the future, we are planning to collaborate with some biomedical labs to validate the practicality of the proposed algorithm.

## Data availability statement

The original contributions presented in the study are included in the article/supplementary material, further inquiries can be directed to the corresponding author.

## Author contributions

FJ, NA, and SA-m performed the main concept and experimental work. WM and MA were made critical revisions, reviewed, help in writing, analysis of this paper, and approved the final version. All authors contributed to the article and approved the submitted version.

## Conflict of interest

The authors declare that the research was conducted in the absence of any commercial or financial relationships that could be construed as a potential conflict of interest.

## Publisher's note

All claims expressed in this article are solely those of the authors and do not necessarily represent those of their affiliated organizations, or those of the publisher, the editors and the reviewers. Any product that may be evaluated in this article, or claim that may be made by its manufacturer, is not guaranteed or endorsed by the publisher.
